# Validity and reliability of the Arabic version of the "Personal Wellbeing Index-Adults" on adults with hearing impairment

**DOI:** 10.34172/hpp.2020.39

**Published:** 2020-07-12

**Authors:** Nesma Ahmed Lotfy

**Affiliations:** Biostatistic Department, High Institute of Public Health, Alexandria University, Egypt

**Keywords:** Validity, Reliability, Quality of life, PWI-A, Hearing loss

## Abstract

**Background:** The Personal Wellbeing Index-Adults (PWI-A) is the most widely used instrumentfor measuring subjective-quality of life (QoL). The current study seeks to investigate the constructvalidity and reliability of the Arabic version of the PWI-A on adults with bilateral hearingimpairment by comparing the single-factor solution with the two-factor solution.

**Methods:** A cross-sectional study was conducted at the Audio-Vestibular Medicine Unit of Alexandria University from July-2017 to January-2018. A total of 205 adults were interviewed tomeasure the subjective-QoL using the PWI-A instrument. Internal consistency was determinedusing both Cronbach’s alpha and composite reliability (CR). Validity was assessed by constructvalidity, including ordinal regression, ordinal exploratory factor analysis (OEFA), and ordinalconfirmatory factor analysis (OCFA).

**Results:** The first four items of the PWI-A which are: satisfaction with living standard, health,achievements, and relationships were the most important indicators of subjective-wellbeing(Part r^2^ 0.0547, 0.0324, 0.0361, and 0.0225, respectively). OEFA suggested that the two-factormodel contributes better than the single-factor model. OCFA validated this suggested solution;(two-factor: RMSEA=0.084 (90% CI=0.01-0.14); CFI=0.964; AIC=52.64; single-factor: RMSEA=0.119 (90% CI=0.07-0.17); CFI=0.922; AIC=62.77). Good internal consistency wasalso presented (two-factor: Cronbach’s alpha=0.719, 0.693; single-factor: Cronbach’s alpha =0.750).

**Conclusion:** The Arabic version of the PWI-A is a multidimensional scale that consists of twodimensions: the first is related to subjective-QoL, and the second is related to satisfaction withthe community. Thus, it is recommended to use the short version of the PWI-A with only fouritems to measure subjective-QoL, as it achieved sufficient reliability and construct validity.

## Introduction

Hearing impairment (HI) has been referred to as an unseen disability and a silent disorder.^1^ It is considered one of the most common types of sensory deficiency in humans which could be partial or total impairment in one or both ears.^2,3^ The World Health Organization (WHO) reported that HI is a worldwide health issue since more than 5% of the world’s population live with this disorder.^3^ It was estimated to be the third most common disability in 2008, and as such, it represents a substantial burden on society.^4^ In 2007, a national household survey was carried out in Egypt to estimate the prevalence of HI, which was reported as 16.0%.^5^

The WHO defined the quality of life (QoL) as “individuals’ perception of their position in life in the context of the culture and value systems in which they live, and in relation to their goals, expectations, standards, and concerns.”^6^ Some studies have demonstrated that HI reduces QoL and interferes with the people’s overall life, relationships, emotional well-being, and feeling of safety.^7,8^

Assessment of QoL among people with HI can be addressed through several instruments such as the Hearing Handicap Inventory for Adults, which is composed of emotional and socio-situational subscales.^9^ Satisfaction with life is considered a vital indicator of QoL. Therefore, the Personal Wellbeing Index–Adults (PWI-A) is the most used instrument worldwide in identifying how individuals feel regarding their lives.^10^ It includes seven satisfaction components, each related to a domain in QoL and was created from the worldwide question “How satisfied are you with your life as a whole?”^11^

Psychometric properties of the PWI-A instrument had been evaluated in different languages.^11^ A good psychometric property was shown in Brazil (reliability; Cronbach’s alpha = 0.796) and Chile (reliability; Cronbach’s alpha = 0.779).^12^ In addition, the PWI-A was found to have an adequate validity and reliability in previous studies of adults in both Australia^11^ and China.^13,14^

Many studies had measured the validity of the PWI-A using the criterion that each domain must contribute a unique variance when all domains are regressed against “Satisfaction with life as a whole.”^11^ It was noticed that the last three domains in some studies had the least contributions.^11^ Thus, we can hypothesis that the instrument consists of two dimensions rather than one dimension, which is subjective QoL. Therefore, the present study assessed the validity and reliability of the Arabic version of the PWI-A on adults with bilateral HI through the comparison of the single-factor solution with the two-factor solution.

## Materials and Methods

### Study design and sample characteristics

A cross-sectional study was carried out from July 2017 to January 2018 at the output patient clinic of Audio-Vestibular Medicine Unit in Alexandria University, Egypt. Individuals audiologically evaluated with bilateral HI and without complete deafness were invited to participate in the study. When the participants were already experience difficulty in hearing properly, talking close to such participant’s ears were sufficient to continue on with the interview.

### Sample size

Based on the rule of thumb for exploratory factor analysis (EFA) and confirmatory factor analysis (CFA), Jöreskog and Sörbom^15^ suggested that, it is best to have a minimum of 10 participants per parameter estimated. However, guidelines as low as 5 to 10 observations per parameter have also been suggested by Floyd and Widaman.^16^ The minimum required sample size was 140 as the PWI-A consists of seven items and two different techniques (ordinal exploratory factor analysis [OEFA] and ordinal confirmatory factor analysis [OCFA]) will be used for validation. However, 205 individuals were enrolled.

### Data collection methods and tools

A pre-designed structured questionnaire was created to include two parts:

- Part I was designed to collect data about the socio-demographic characteristics of the study sample. This included: sex, age, marital status, educational level, and occupation.
- Part II included the PWI-A instrument, which is comprised of seven satisfaction components, each one assessing a domain in QoL. The following components included are living standard, health, achievement, relationships, safety, community connectivity, and future security. Each domain is rated on a 3-point Likert scale (1= Not satisfied, 2 = Moderately satisfied, 3 = Satisfied). Overall satisfaction was also assessed by a global question, “How satisfied are you with your life as a whole?” which reflects the individual’s general life satisfaction (GLS) ^11^ (Supplementary file 1). Regarding the PWI-A scoring, each of the seven domains can be analyzed as a separate variable, or the seven domain scores can be summed up to get an average score, which reflects “Subjective Wellbeing”.

The PWI-A was translated into the Arabic language using a forward and backward method performed by three experts. First, one of the experts translated it into Arabic. Next, the Arabic version was translated back into the original language by another expert. Finally, the three experts compared the two versions and presented the final version of the Arabic PWI-A.

Face validity was assessed through a pilot study to check the applicability of the scale (grammar, organization, and appropriateness). Content validity was examined by three experts. These experts were requested to determine the essence of each item using a 3-point Likert scale (essential, not essential, and useful but not essential). All experts agreed that all items were essential. The panel of experts hailed from the High Institute of Public Health in Alexandria University. Discriminant validity was performed to examine the applicability of the PWI-A among literate and illiterate individuals. Such was done by discriminating between the scores of the first quartile (≤Q1) and the third quartile (≥Q3), using the Mann-Whitney test. For literate individuals (n = 100), the first quartile (Median (IQR) = 11(3)) and the third quartile (Median (IQR) = 19(2)) of the PWI-A scores were statistically significant (Z = -6.813, *P* = 0.000). Similarly, for the illiterate individuals (n = 95), the first quartile (Median (IQR) = 10(2)) and the third quartile (Median (IQR) = 18(2)) of the PWI-A scores were statistically significant (Z = -6.539, *P* = 0.000).

### Statistical analysis

*Data management*

The data were collected over a period of six months. The collected data were checked for integrity and completeness. They were then, coded and fed to a computer software. The statistical package for the social sciences (IBM^®^ SPSS^®^ Statistics version 25.0) was used for data entry and descriptive analysis.

*Data analysis*

Descriptive statistics were calculated using a mean ± standard deviation (SD) or frequency and percent, where it is appropriate. The highest and lowest total scores were used to define the ceiling and floor effect, a percentage of more than 15% was considered for detecting the effect.^17^ In the current study, the floor effect was 1% while the ceiling effect was 4.4%. Correlations between the PWI-A items were calculated using Spearman’s rank correlation. Construct validity was assessed using three methods: (i) calculation of the unique and shared variance for the PWI-A items, (ii) OEFA, and (iii) OCFA.

Ordinal regression was performed to measure the adjusted R^2^ Nagelkerke, which describes the total explained variance of the PWI-A items on the GLS. The unique variance of each of the seven items as against the GLS was determined by squaring the semi-partial (part) correlation based on Spearman’s rank correlations. Statistical R programming was used for this purpose.^18^

OEFA was conducted using “the Proportional Odds Model Approach” (POM).^19^ Two separate exploratory factor analyses were conducted (single-factor, and two-factor models) to understand how the items are loaded on each respective factor. An oblique (promax) rotation was employed in the two-factor model. To check the assumptions for factor analysis; the Kaiser–Meyer–Olkin (KMO) test was used to evaluate the sampling adequacy, and Bartlett’s test of sphericity was performed to confirm the appropriateness of data.

OCFA was performed using weight least squares estimation and polychoric correlations with the asymptotic covariance matrix as a weight matrix in both models.^20^ To assess the model fit, various indices of fit were examined: chi-square, root mean square error of approximation (RMSEA), and its 90% confidence interval (value of 0.05 to 0.08 indicates a close fit) as an absolute fit index, adjusted goodness-of-fit index (AGFI), comparative fit index (CFI), and normed fit index (NFI) (with values close to 0.90 or 0.95 reflecting a good model fit) as incremental indices. The Akaike information criterion (AIC) was used to compare various factor structures. Lower AIC values would indicate a better fit.

The collected data were randomly split into two datasets, one for conducting OEFA (n = 100) and the second for validation using OCFA analysis (n = 105). The OEFA and OCFA were run using the LISREL version 8.8 software.^21^

Two different techniques were utilized to measure internal consistency. The first is Cronbach’s alpha, while the other is composite reliability (CR) which based on ordinal confirmatory factor loading (a value of CR > 0.6 indicated a good CR for a construct).

## Results

### Characteristics of the study population

In total, 60.5 % of the samples were females, the ages ranged from 18 to 65 years with a mean age of 42.33 ± 14.58 years. The majority of the enrolled adults were married (69.8%), unemployed (63.0%) and illiterate (46.3%) (Table 1). According to the PWI-A, 56.6% of the participants reported that they felt safe, 47.4% admitted satisfaction with their personal relationships, and 38% were satisfied with their achievements. Moreover, the mean average score was 2.08, SD = 0.52, skewness = -0.126, and kurtosis = -0.92 (Table 2).

**Table 1:** The socio-demographic characteristics of enrolled adults with hearing impairment

**Socio-demographic characteristics**	**Total participants** **(n = 205)**	
	**No.**	**%**
Sex		
Male	81	39.5
Female	124	60.5
Age (y)		
18-	46	22.4
30-	84	41.0
50-65	75	36.6
Min-Max	18 – 65	
Mean ±SD	42.33 ± 14.58	
Marital status		
Single	37	18.0
Married	143	69.8
Divorce	6	2.9
Widow	19	9.3
Education		
Illiterate	95	46.3
Low (primary and preparatory)	49	23.9
Middle (secondary, technical diploma)	49	23.9
High (university degree)	12	5.9
Occupation		
Not working	129	63.0
Official work	31	15.1
Unofficial work	29	14.1
Pension	16	7.8

**Table 2:** Personal Wellbeing Index - Adults (PWI-A) in the study sample

**Items**	**Total participants (n = 205)**		
	**Not Satisfied** **(%)**	**Moderately Satisfied (%)**	**Satisfied** **(%)**
PWI1: living standard	42.4	31.2	26.4
PWI2: health	16.1	51.2	32.7
PWI3: achievement	25.4	36.6	38.0
PWI4: relationships	30.2	22.4	47.4
PWI5: safety	30.7	12.7	56.6
PWI6: community connectivity	31.7	24.4	43.9
PWI7: future security	39.0	30.3	30.7
Mean ± SD	2.08 ± 0.52		
Kurtosis and skewness	-0.92, -0.126		

### Construct validity 

Spearman’s rank correlation among the seven items of the PWI-A and the GLS ranged from 0.131 to 0.61. The total explained variance on overall satisfaction was 60.9%. The unique contribution of standard of living to the total explained unique variance was 5.47%. The seven items contributed 15.1% in unique variance. This means that the shared variance between items was 45.8%. In addition, three items had a negligible contribution to the explained unique variance. These items included: safety, future security and community connectedness (Table 3).

**Table 3:** Spearman’s rank correlation between the seven items of the Personal Wellbeing Index – Adults (PWI-A)

	**PWI**	**PWI1**	**PWI2**	**PWI3**	**PWI4**	**PWI5**	**PWI6**	**PWI7**	**Part r** ^ 2 ^
PWI: GLS	1								
PWI1: living standard	0.610**	1							0.0547
PWI2: health	0.581**	0.555**	1						0.0324
PWI3: achievement	0.536**	0.475**	0.427**	1					0.0361
PWI4: relationships	0.455**	0.315**	0.397**	0.283**	1				0.0225
PWI5: safety	0.208**	0.210**	0.196**	0.154*	0.261**	1			0.0005
PWI6: community connectivity	0.218**	0.134	0.131	0.181**	0.280**	0.357**	1		0.0009
PWI7: future security	0.347**	0.292**	0.250**	0.285**	0.363**	0.485**	0.466**	1	0.0036
Total explained unique variance	0.151								
Adj R2 (Nagelkerke)	0.609								
Total explained shared variance	0.458								

* Correlation is significant at the 0.01 level (2-tailed); ** Correlation is significant at the 0.05 level (2-tailed).

### Exploratory factor analysis

Ordinal EFA was conducted (n = 100) on the seven items of the PWI-A. The KMO verified the sampling adequacy [KMO = 0.754 (fair)], while the Bartlett’s test of sphericity confirmed the factorability of the correlation matrix (χ² (21) = 183.236, *P* < 0.001).

The single-factor model revealed that all the items loaded well. However, items 2, 1, 4, and 3 had the smallest factor loadings, respectively. In the two-factor model, items 1,2,3, and 4 loaded better on the first factor while items 5, 6, and 7 loaded better on the second factor (Table 4). Items 2 and 7 had a standardized factor loading value greater than one, since the factor loading is a regression coefficient which can exceed one.^22^

**Table 4:** Ordinal Exploratory Factor Analysis (OEFA) for the Personal Wellbeing Index - Adults (PWI-A).

**Items**	**Item-factor loadings**		
	**Single-factor**	**Two-factor**	
		**Factor one**	**Factor two**
PWI1: living standard	0.706	0.838	0.096
PWI2: health	0.573	1.036	-0.272
PWI3: achievement	0.721	0.773	0.164
PWI4: relationships	0.718	0.589	0.326
PWI5: safety	0.92	-0.072	0.94
PWI6: community connectivity	0.849	0.008	0.846
PWI7: future security	0.965	-0.024	1.003
Kaiser-Meyer-Olkin Measure	0.754		
Bartlett's Test of Sphericity	Approx. chi-square =183.236, *df* =21, *P* <0.001		

### Confirmatory factor analysis

An OCFA was performed after OFA to generalize the result on the validation sample. The results of single-factor model as against the two-factor model are displayed in Table 5. The single-factor model represents fitting with chi-square = 34.77, df =14, *P* = 0.001; RMSEA = 0.119 (90% CI = 0.07-0.17); AGFI = 0.932; CFI = 0.922; NFI = 0.879; AIC = 62.77. However, the two-factor model had a better fitting; chi-square = 22.64, df = 13, *P* = 0.046; RMSEA = 0.084 (90% CI = 0.01-0.14); AGFI = 0.952; CFI = 0.964; NFI = 0.92; AIC = 52.64.

**Table 5:** Ordinal Confirmatory Factor Analysis (OCFA) for the Personal Wellbeing Index – Adults (PWI-A)

**Model**	**χ 2 (** ***P*** **value)**	**RMSEA (90% CI)**	**(AGFI)**	**(CFI)**	**(NFI)**	**Model (AIC)**
Single – factor	34.77 (0.001)	0.119(0.07, 0.17)	0.932	0.922	0.879	62.77
Two – factor	22.64 (0.046)	0.084 (0.01, 0.14)	0.952	0.964	0.92	52.64

**Figure 1:**
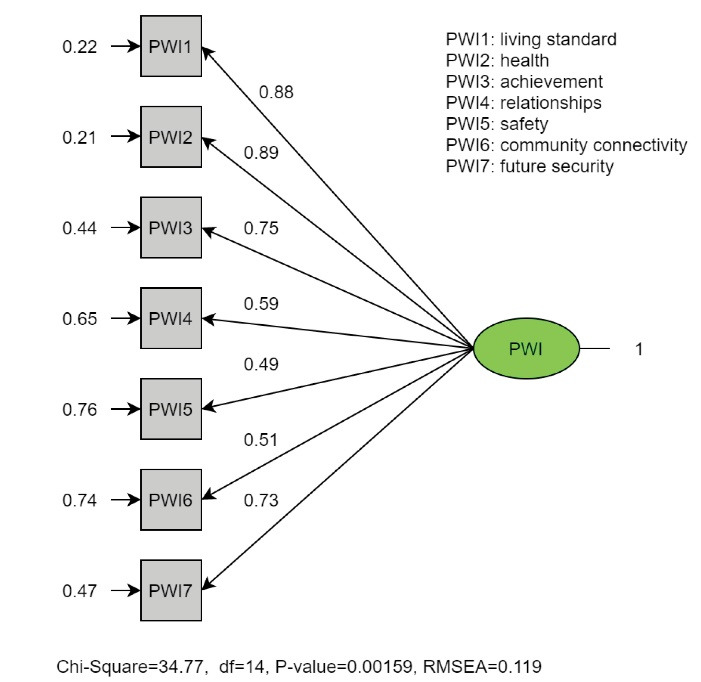
The standardized parameter estimates of the ordinal confirmatory factor analysis of the Personal Wellbeing Index – Adults (PWI-A) for the single-factor model.

**Figure 2:**
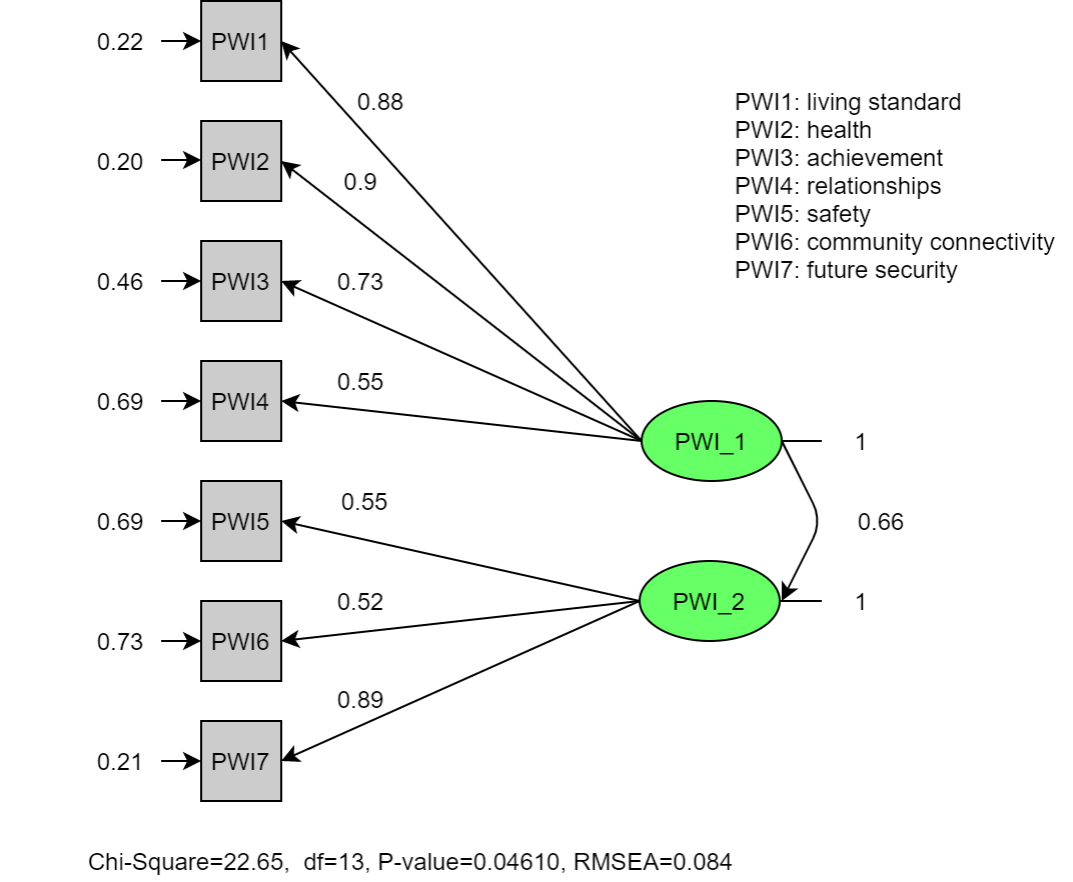
The standardized parameter estimates of the ordinal confirmatory factor analysis of the Personal Wellbeing Index – Adults (PWI-A) for the two-factor model.

Figures 1 and 2 show the entire standardized factor loading for the two models (single-factor model versus two-factor model). With regard to the second model, the loading of PWI5 and PWI6 items had slightly improved, while the last item (PWI7) had dramatically increased from 0.73 to 0.89. Moreover, the error term of PWI7 had dramatically decreased from 0.47 to 0.21. The highly correlated error in the two-factor model may be the result of similar expressions used in the instrument.

### Reliability

The CR and Cronbach’s alpha values for the PWI-A were almost equal or greater than 0.7, indicating good reliability (Table 6).

**Table 6:** Reliability of the Personal Wellbeing Index - Adults (PWI–A)

**Cronbach’s alpha if Item deleted**	**Single-factor**	**Two- factor**	
		**Factor one**	**Factor two**
PWI1: living standard	0.717	0.622	-
PWI2: health	0.718	0.615	-
PWI3: achievement	0.722	0.664	-
PWI4: relationships	0.718	0.728	-
PWI5: safety	0.730	-	0.627
PWI6: community connectivity	0.736	-	0.641
PWI7: future security	0.697	-	0.532
Cronbach’s alpha	0.750	0.719	0.693
Composite reliability	0.870	0.856	0.701

## Discussion

HI can reduce the QoL because it influences one’s overall life, relationships with other people, and communication. Therefore, using an easy and short instrument for measuring QoL is necessary for considering their disability. Furthermore, the PWI-A is the easiest instrument that can measure subjective-QoL because it consists of only seven items. Several studies have tested the validity of the PWI-A in various countries, such as in Argentina, China, and others,but not in Egypt.^11^ Thus, this research will serve as a guideline for the PWI-A international well-being group.

Considering the contribution of each item on the GLS, the results were in line with van Beuningen and DeJonge’s who found that the first four items of the PWI-A (satisfaction with living standard, health, achievements, and relationships) had the highest unique variance contribution (0.15, 0.03, 0.04, and 0.07, respectively). However, the last three items had the lowest contributions (0.00, 0.00, and 0.00, respectively).^23^ A similar conclusion was drawn in several studies from different countries.^11,24^ Likewise, the total explained variance on overall QoL was 66% in the Netherlands,^23^ 54% in the United States,^24^ and 60.9% in Egypt. Moreover, three studies assessed the validity in Argentina, with a total explained variance of 35%, 39%, and 57%, respectively.^11^

The major contributor item on overall satisfaction (GLS) varies between countries. Life achievement was the dominant item in the United States (unique variance = 11%),^24^ while the living standard was the dominant item in the Netherlands^23^ (unique variance = 15%) and Egypt (unique = 5.47%). We can conclude that every community has a different domain of satisfaction that may result from different characteristics of each country. These characteristics include: economic status, culture, and healthcare systems.

EFA was used to explore the PWI-A structure among gifted college students in the United States. A two-factor extraction that explaining approximately 60% of the variance was found. The first factor contained community connectedness, personal relationships, achievements in life, and future security. The other factor had personal safety, standard of living, and health.^24^ Another study had conducted factor analysis to examine the validity of the PWI-A among mothers of mentally retarded students in the North of Tehran, Iran. They reported that the PWI-A contributes to only one factor, which was subjective-QoL.^10^ The Netherlands had the same conclusion as Iran, with the factor loading of the factor analysis for the seven items as 0.81, 0.72, 0.81, 0.73, 0.65, 0.78, and 0.79, respectively.^23^ However, in this present study, the PWI-A contributed to the two-factor model better than the single-factor model (two-factor loading: factor-one: 0.838, 1.036, 0.773, 0.589, factor-two: 0.94, 0.846, 1.003). From this we can realize that the last three items, which are satisfaction with safety, community connectivity, and future security do not contribute to subjective-QoL since 56.6% of participants felt safe, 31.7% felt isolated from community, and 39.0% unsatisfied regarding future security.

A sample of 1965 participants forming Survey 22 of the Australian Unity Wellbeing Index^25^ were acquired to conduct CFA. CFA suggested that the PWI-A fit adequately in a uni-dimensional construct (chi-square = 139.43, *P* < 0.000, CFI = 0.96, RMSEA = 0.08) despite the difference between age, and gender across samples.^26^ In contrast, a single-factor model and two-factor model were performed using CFA in the USA,^27^ the single-model had an acceptable CFI (0.956), but a high RMSE (0.12, 95% CI = 0.1-0.14). On the other hand, the two-factor model (factor one: standard of living, health, achievement, and safety; factor two: relationships, community connectivity, future security) yield a poor model fit (RMSE > 0.08, CFI <0.95).^27^ In this study, the OCFA validated the results suggested by the OEFA. The two-factor model had a more adequate fit rather than the single-model (AIC = 52.64 and 62.77, respectively, RMSE = 0.084 (90% CI = 0.01-0.14), and 0.119 (90% CI = 0.07-0.17), respectively, and CFI = 0.964, and 0.922, respectively).

The PWI-A had good reliability in previous studies of adults in Australia and other countries, ranging from 0.7 to 0.85.^11^ In Egypt, the Cronbach’s alpha for the single-factor model was 0.75, while for two-factor model, it was 0.719 and 0.693. Since the two-factor model had a small number of items in each (factor one = four items, factor two= three items), the Cronbach’s alpha had slightly decreased compared to the single-factor model.^28^

The present study has some limitations. Most of the participants were women, illiterate, and unemployed. Moreover, the Likert scale used here was 3-point Likert-scale while the original version of the PWI-A utilized a 10-point Likert-scale. Because of this, abroad option for participants was not achieved. Consequently, further studies with the male population, and employed individuals using a 10-point Likert-scale is needed to generalize our findings.

## Conclusion

The Arabic version of the PWI-A is a multidimensional scale that consists of two dimensions: the first one related to subjective-QoL and the second related to satisfaction with community. Thus, it is recommended to use the short version of PWI-A with only 4-items to measure the subjective-QoL among adults with HI, as it achieved sufficient reliability and construct validity.

## Availability of data and materials section

All data are available from the corresponding author upon responsible request.

## Ethical approval

The study was approved by the Institutional Review Board and the Ethical Committee of High Institute of Public Health in Egypt. Verbal consent was taken from the study participants after an explanation of the purpose and benefits of the research. Anonymity and confidentiality of the participants’ data was ensured and maintained.

## Competing interests

The author declares that there are no competing interests.

## Funding

There was no source of funding.

## Acknowledgments

The author would like to thank Dr. Hesham Kozou, Professor of Otorhinolaryngology, Faculty of Medicine, Alexandria University (Egypt), for facilitating the recruitment of the study participants and data collection.

The author is grateful as well to Dr. Mona Hassan, Professor of Biostatistic, High Institute of Public Health, Alexandria University (Egypt), and Dr. Ekram Abd El-Wahab, Associate Professor of Tropical Health, High Institute of Public Health, Alexandria University (Egypt), for the critical appraisal of the manuscript. The participants of the study are also highly appreciated.

## Supplementary Materials


Supplementary file 1 contains PWI-A instrument.
