# Supplementary material for: Validity and reliability of the Arabic version of the "Personal Wellbeing Index-Adults" on adults with hearing impairment
Source: Health Promot Perspect. 2020 Jul 12;10(3):250–6. doi: 10.34172/hpp.2020.39 (PMC7420167; doi:10.34172/hpp.2020.39)
Supplement: Supplementary file 1 — contains PWI-A instrument. [file hpp-10-250-s001.pdf]

Supplementary file 1. Personal Wellbeing Index - Adult (PWI –A)

	**Satisfied (3)**	**Moderately satisfied (2)**	**Not satisfied (1)**
1- How satisfied are you with your standard of living?<br>إلى أي مدى انت راضى بمستوى معيشتك ؟			
2- How satisfied are you with your health?<br>إلى أي مدى انت راضى بحالتك الصحيه ؟			
3- How satisfied are you with what you are achieving in life?<br>إلى أى مدى انت راضى عن ما انجزته او حققته فى حياتك ؟			
4-How satisfied are you with your personal relationships?<br>إلى أي مدى انت راضى بعلاقاتك الشخصية؟			
5-How satisfied are you with how safe you feel?<br>إلى أي مدى انت راضى بشعورك بالامان ؟			
6- How satisfied are you with feeling part of your community?<br>إلى أي مدي انت راضى بأنك تشعر بالانتماء الى مجتمعك او انك جزء منه؟			
7- How satisfied are you with your future security?<br>إلى اى مدى انت راضى بالامن حول مستقبلك؟			

General life satisfaction (GLS)

"Thinking about your own life and personal circumstances, how satisfied are you with your life as a whole?"

استناداً إلى حياتك الشخصية وظروفك الشخصية، إلى أي مدي تشعر بالرضى عن حياتك بشكل عام ؟

Satisfied (3)
Moderate (2)
Not satisfied (1)
